# Point of care obstetric ultrasound training for midwives and nurses: implementation and experiences of trainees at a rural based hospital in Sub-saharan Africa: a qualitative study

**DOI:** 10.1186/s13104-023-06569-8

**Published:** 2023-10-24

**Authors:** Aloysius G. Mubuuke, Geoffrey Erem, Rita Nassanga, Elsie Kiguli-Malwadde

**Affiliations:** 1 Radiology Department, School of Medicine, Makerere University, Kampala, Uganda; 2 Health Workforce and Development, African Centre for Global Health and Social Transformation, Kampala, Uganda

**Keywords:** Point of Care Obstetric Ultrasound, Nurses, Mid-wives, Rural, Uganda

## Abstract

**Background:**

Point of care ultrasound training has been successfully implemented in some settings. This has been done due to a shortage of radiology human resource gap especially in the rural areas of low-resource settings. The purpose of the study was to implement a point of care obstetric ultrasound training program for midwives and nurses and explore their experiences following the training at a rural based hospital in Uganda.

**Methods:**

It was an exploratory qualitative study with some elements of implementation research design involving midwives and nurses that had undergone obstetric ultrasound training at Kiwoko hospital, a rural-based hospital in Uganda. Purposive sampling was used to select twenty-five midwives and nurses. These participants underwent a 6-weeks training in point of care obstetric ultrasound. Following the training, in-depth interviews were conducted to obtain the experiences of the participants.

**Results:**

The training was conducted by qualified radiologists and sonographers and it involved both didactic sessions and rigorous practical and clinical demonstrations and eventually real-time scanning of the women. Three key themes emerged from the interviews: (1) Gaining important obstetric ultrasound skills, (2) Improving management of pregnant women and (3) Positive for task-shifting.

**Conclusion:**

The point of care obstetric ultrasound training program was successfully implemented at Kiwoko Hospital. The trainees reported positive experiences from the training and while only conducted at one rural health facility, the overwhelmingly positive experience from trainees underscores the importance of point of care obstetric ultrasound in delivering imaging services.

**Supplementary Information:**

The online version contains supplementary material available at 10.1186/s13104-023-06569-8.

## Background

Point of care ultrasound (POCUS) is the practice of medical professionals using ultrasound to make quick clinical decisions [1]. POCUS has been implemented in many settings with great success [2–8]. The aim is task shifting where key basic ultrasound examinations that would inform immediate clinical decisions can be performed by a trained health worker who may not necessarily be a radiologist or sonographer. POCUS brings ultrasound services closer to the people to aid in diagnosis and contribute to quick patient management without waiting for referrals to tertiary health facilities [9]. This may eventually contribute to a reduction in many preventable deaths. In Uganda where this study took place and indeed largely in many low and moderate-income countries (LMICs), there are few radiologists and sonographers who are trained formally to provide ultrasound services, yet equipment is available and patient numbers in need of obstetric ultrasound services is high [10–14], hence the need for POCUS training. Obstetric ultrasound has also become part of routine antenatal care and mothers move long distances to access it due to the limited numbers of radiology professionals in rural communities. The idea of equipping other cadres of health workers with basic skills to perform point of care ultrasound services especially in emergency situations that require immediate clinical intervention is thus paramount to reduce maternal mortality.

In Kenya, a novel POCUS training program was introduced [6]. Researchers of this study recommended focused trainings with well-defined outcomes as a way of increasing the competency of non-imaging professionals in using ultrasound. Other POCUS training programmes have reported similar success [4, 5, 9]. Despite the fact that Uganda has started advocating for such training programs to be introduced especially for rural-based mid-wives and nurses where there is a clear shortage of radiology professionals, there is a dearth of published literature from our settings reporting the design and implementation of such training programs.

Maternal mortality due to pregnancy-related complications remains high in many LMICs [15]. Many of the obstetric complications that result into maternal deaths could be prevented through early detection of such complications using ultrasound [16]. However, in many settings, there are few radiologists and sonographers that can ably perform obstetric ultrasound which may lead to maternal mortality through delayed referrals for obstetric ultrasound services. Task-shifting through training midwives and nurses the basic obstetric ultrasound skills would alleviate a number of such deaths through identification of cases that require immediate attention without waiting for a qualified radiologist/sonographer or referring the patient to distant health facilities where qualified personnel are located. There is also limited evidence of how focused training and role-extension of obstetric ultrasound services to other health workers can address this challenge in the setting of limited human resource. The purpose of this study therefore was to design and implement a POCUS training programme for rural based nurses and mid-wives and then explore experiences of the trained nurses and midwives about the point of care obstetric ultrasound.

## Methods

This was an exploratory qualitative study that aimed at implementing a POCUS training programme for mid-wives and nurses and explore their experiences of this intervention. Thus the study also employed aspects of an implementation research design to develop a POCUS training curriculum, implement and assess the experiences of the trainees. The study including the training was conducted at Kiwoko hospital, a rural based hospital in Uganda. The targeted participants were nurses/mid-wives. In order to select the participants, purposive convenience sampling was used to select twenty-five nurses/mid-wives. Purposive sampling is valuable in qualitative studies such as this as they target those participants with the characteristics and qualities needed to address the research aim. In this study, the midwives and nurses were the ones targeted and those present and willing to participate at the time of the study were thus recruited, hence the convenience sampling approach as well.

### The POCUS curriculum

A POCUS curriculum was developed in focused obstetric ultrasound by a panel of experts who agreed on the final competencies. The experts included 4 radiologists and 3 sonographers. The key competencies that were targeted included: identification of a fetus, identification of number of fetuses, identifying a fetal heartbeat, demonstrating fetal lie and presentation, localizing the placenta, assessing maternal cervical length, assessing amniotic fluid adequacy, estimating fetal age using MSD, CRL & BPD and demonstrating professionalism and ethics while scanning. The training was for 6 weeks and had both didactic and practical sessions.

### Qualitative interviews following the training

At the end of the training, twenty-five interviews were conducted with the trainees to explore their experiences of the training. Responses from the interviews were audio-recorded, transcribed and thematic analysis conducted. The questions asked during the interviews were about the experiences of the trainees about the obstetric ultrasound training, the skills gained, how the skills gained would be utilized and their attitude towards being trained in point of care obstetric ultrasound (Supplementary Material 1). These questions were first piloted with three midwives who do not work at Kiwoko hospital where this study took place from. The interviews were conducted by the research assistants till data saturation was attained, audio-recorded and later transcribed. Rigor and trustworthiness of the study including credibility, conformability, dependability and transferability were ensured by detailing the research process, member checking of the transcripts and documenting every step taken through the research process. The transcripts were also reviewed against the audio-recordings to ensure that the right message had been obtained. Thematic analysis was inductive in nature where the transcribed data was read several times to identify patterns of meaning called codes. The codes were again related to each other to form categories and the eventual themes that have been used to report the findings.

### Ethics

Approval to conduct the study was granted by Makerere University School of Health Sciences Research Ethics Committee (REC No. 2019-080).

## Results

### Socio-demographic characteristics

Of the twenty-five participants, five were nurses, fifteen were midwives and five were nurse/midwives. Five of them were males while twenty were female. None of them had prior ultrasound training.

### Qualitative interviews

Three themes were generated including: (1) Gaining important obstetric ultrasound skills; (2) Improving management of pregnant women and (3) Positive attitude for task-shifting.

### Theme 1: gaining important Obstetric Ultrasound skills

The participants demonstrated satisfaction and competence in the obstetric ultrasound skills they had gained which involved scanning pregnant women to identify key obstetric indicators of either good or poor outcome. The skills gained seemed to be valuable as part of their daily clinical work and they reported that such skills are more likely to improve their clinical duties.

“*As a midwife, it was extremely important for me to gain some knowledge and skills in scanning pregnant mothers. Sometimes we do our work with a lot of challenges, but having these skills to evaluate some of the basic things like how many babies are there, how the baby is presenting is good for us…. there is no need to disturb mothers to go to town to look for scans when we can now do some of these here*”.

“*We have had these machines here, but not knowing how to use them, so we tell mothers to go to town. From this training, I have realized that some key things like me knowing how the placenta is, if water around the baby is enough, if baby`s heart is beating can be done by myself without waiting for a radiology person or telling the mother to go to town to scan. I can just use the machine here to evaluate these and know what to advise the mother*”.

From the above responses, one can deduce that the participants gained some valuable skills which they believe can be utilized to enhance their daily clinical practice.

### Theme 2: improving management of pregnant women

One other important aspect that came up related to utilizing the skills gained to improve the management and handling of pregnant women. Most of the responses contextualized the situation at the antenatal clinics and obstetric wards in which it could be observed from the responses that when midwives and nurses gain some of the skills needed to examine pregnant women using ultrasound, their patient management is likely to improve in the long run:

“*I think when us who first encounter and manage these mothers get these skills, we shall handle and advise our mothers better. Using the ultrasound machine as a midwife can give me more clues on how to handle a particular mother and see if she needs to see a more qualified person or if she does not*”.

“*The mothers will be managed better. If I get to know that the baby is lying in a breech way, I then know how to handle that particular mother better. I can now use the machine to check how the baby is lying when compared to before here sometimes you would not be sure and tell the mother to first go and do a scan and come back. Doing it myself to quickly check this surely improves and quickens the time she spends in the hospital and we can work on her very fast*”.

From the responses above, one can infer that when midwives/nurses are able to use the ultrasound machines to quickly assess some key parameters during the course of their duty, it not only improves management, but could also shorten the time of hospital stay for the mothers.

### Theme 3: positive attitude for task-shifting

All trainees reported that training in some of the basic obstetric ultrasound competencies is good for task-shifting since the midwives and nurses directly handle the management of pregnant women. It was noted that responses under this theme leaned more towards the fact that radiologists and sonographers who are formally qualified to perform obstetric ultrasound are still few in number and majority are concentrated in large urban hospitals. The following responses reflect this observation:

“*I think this kind of training is timely and hopefully it could be made more frequent and also accessible to all hospitals in rural areas because these have no radiology staff to do the scans. We refer women to go to towns in private clinics to do the scans yet some of them can be performed by us the midwives and make some quick decisions*”.

“*After undergoing this training, I can now do some basic things like identifying number of babies, looking for heartbeat, seeing the placenta, know how the baby is presenting and water around the baby. We used to refer the women to radiology to do even these. It is good that some of these basic checks are done by us to help us make quick clinical decisions. Some centers have these machines, but no one can use them yet mothers would benefit from them….training us to do some of these basic but important spot checks would save our women a lot of trouble*”.

## Discussion

This study set out to implement a POCUS training involving midwives and nurses at a rural based health facility in Uganda and then explore the experiences of the trained midwives/nurses about the point of care obstetric ultrasound. From the findings, the training was beneficial and participants reported positive experiences that rotated around the importance of gaining skills in obstetric ultrasound, improving management of pregnant women and the aspect of role extension to them. This findings is in resonance with previous literature which has reported the importance of non-radiology health professionals gaining key competencies in POCUS [1, 2, 6]. It should be noted that many of the midwives/nurses in LMICs are the anchors of the primary health care system and usually interface with mothers first during antenatal care. Therefore, they are the initial primary contact when mothers report for antenatal care and indeed perform the initial maternal assessments and examinations from which they make their clinical judgments on whether to refer the mother or not to a more qualified professional such as a doctor. As a point of care assessment tool, ultrasound can provide an optimum tool for the midwives or nurses to make quick check ups and assessment of the pregnant women before deciding any further management for such mothers.

Using ultrasound seemed to ease the work of the midwives/nurses as it provided a quick way of assessing basic obstetric parameters such as number of fetuses, fetal presentation and lie, placental position and evaluation of adequacy of the amniotic fluid volume, parameters which would have otherwise led to more referrals and hence cause more delay in attending to the women. Ordinarily, these would take time perhaps waiting for radiologists or sonographers, but using ultrasound by the trained midwives and nurses seemed to have accelerated the process and hence reducing hospital stay. Therefore, POCUS in obstetric assessment can be incorporated into routine practice for midwives/nurses to make quick clinical decisions and judgments without first referring mothers to private health facilities. Previous studies support this finding in settings where POCUS has been evaluated for non-radiology professionals [1, 4].

In many LMICs, there are limited qualified radiologists and sonographers when compared to patients that require obstetric ultrasound care [10, 11], and majority of these are concentrated in urban areas when compared to rural areas [12, 17]. Therefore, there is need for POCUS training where health care workers such as midwives and nurses that interface with the pregnant mothers are equipped with skills that enable them to make quick clinical decisions using ultrasound. This is likely to assist mothers especially in rural health facilities to receive a service, identify emergency cases, shorten duration of hospital stay and reduce unnecessary referrals. In the short-term, this can potentially off-set the shortage of radiologists and sonographers and also identify high risk cases that require immediate clinical intervention. Despite the importance of point of care obstetric ultrasound where non-radiology professionals perform a limited range of ultrasound skills, it is strongly encouraged that any POCUS services provided by non-radiology professionals should be preceded by training with well-defined outcomes.

In addition, POCUS needs focused regulation. Currently in Uganda where this study was conducted from, there is no formal regulation and supervision for the POCUS trainees outside their formal professional bodies such as the Nurses and Midwives Council. This body is not competent enough to supervise cadres trained in POCUS and this could potentially be the situation in many settings where POCUS has been adopted. For effective implementation and supervision, this needs to be taken care of. Many other settings looking into implementing POCUS should also be cognizant of this such that there is adequate regulation by professional bodies for those non-radiology professionals trained in POCUS. The trained health workers should potentially use their POCUS knowledge to inform their routine clinical decisions in the process of quickly managing pregnant women. Comprehensive obstetric ultrasound investigations remain the preserve of rigorously trained imaging professionals and they should have a hand in supervising those trained under POCUS. We urge the trained people to stick to what they have been trained to do and not to write ultrasound reports. The basic ultrasound skills gained are meant to aid them in performing routine clinical duties in the absence of radiologists or sonographers.

Overall this study has demonstrated that extending basic obstetric ultrasound skills to midwives and nurses can assist in addressing the human resource gap especially in emergency cases that require urgent interventions and quick decisions. If trained, midwives/nurses can ably utilize ultrasound to improve care and management of pregnant women. We thus suggest that many settings with human resource gaps can benefit from such an initiative. However, it also requires regulation and supervision of the trained people so that they do not overstep their mandate. A longitudinal follow up of the performance of the trained midwives/nurses to assess skills retention as well as evaluate impact of POCUS on the health care system overtime were beyond the scope of this work but recommended.

## Limitations

- The study was qualitative in nature involving small numbers of participants and therefore, findings may not be generalizable to very large populations.
- In addition, the study was conducted in only one hospital and thus findings may not necessarily be applicable to other hospital settings.

## Conclusion

The participants reported positive experiences and satisfaction with the skills gained that would enhance their clinical decisions. We do recommend similar programmes in settings with radiology human resource gaps, but emphasis should also be put on regulation and supervision.

### Electronic supplementary material

Below is the link to the electronic supplementary material.

Supplementary Material 1

## Data Availability

Data are available upon request to the corresponding author.
