# Supplementary material for: Point of care obstetric ultrasound training for midwives and nurses: implementation and experiences of trainees at a rural based hospital in Sub-saharan Africa: a qualitative study
Source: BMC Res Notes. 2023 Oct 24;16:287. doi: 10.1186/s13104-023-06569-8 (PMC10598935; doi:10.1186/s13104-023-06569-8)
Supplement: Supplementary file 1 — Supplementary Material 1 [file 13104_2023_6569_MOESM1_ESM.docx]

**Interview Schedule**

**Study Title: Point of Care Obstetric Ultrasound training for mid-wives and nurses: Implementation and experiences of trainees at a rural based hospital in Sub-Saharan Africa: A Qualitative Study**

Qn.1: Share with us your experience of undergoing the point of care obstetric ultrasound training

Qn.2: Comment about the knowledge and skills gained through this point of care obstetric ultrasound training

Qn.3: How did the skills gained help you to improve your clinical work? (*Probe to find out how the routine clinical work has been improved as a result of undergoing training in point of care obstetric ultrasound*)

Qn.4: Specifically, share how the skills gained will influence the care given to the pregnant women who come for antenatal care at the hospital. (*Probe to see how the skills gained have positively impacted pregnant women at the hospital*)

Qn.5: Regarding addressing the shortage of people who do the ultrasound scanning in many rural hospitals, how do you think this training to mid-wives and nurses will address this shortage? Specifically, how do you feel about training mid-wives and nurses in point of care obstetric ultrasound in regard to care for pregnant women? (*Probe to bring out the aspect of task-shifting where mid-wives/nurses can do the basic obstetric scans*)
